# Day-of-Surgery Cancellations in NHS and Independent-Sector Elective Surgery in England: A Narrative Review of Publicly Available Data

**DOI:** 10.7759/cureus.107720

**Published:** 2026-04-26

**Authors:** Muskan Shrivastava, Ish Suri

**Affiliations:** 1 Surgery, North Shore Hospital, Auckland, NZL; 2 Anaesthesia, South Warwickshire Foundation Trust, Warwick, GBR

**Keywords:** cancellation, day-of-surgery cancellation, elective surgery, independent sector, nhs, perioperative medicine, private provider, theatre efficiency

## Abstract

Day-of-surgery cancellation of elective surgery causes patient harm, wastes theatre capacity, and reflects wider perioperative system pressure. In England, the principal public source for routine surveillance is the NHS England Quarterly Monitoring of Cancelled Operations (QMCO) collection, but direct comparison between NHS trusts and independent-sector providers remains methodologically difficult. This revised narrative review synthesised 10 publicly available NHS England datasets/documents and 13 supporting peer-reviewed or policy sources relevant to elective surgery cancellation and independent-sector provision. Publicly available national time-series data show that last-minute non-clinical cancellation rates have generally remained around 1% of elective admissions since the early 2000s, whereas the proportion of cancelled patients not treated within 28 days increased substantially after the COVID-19 pause in data collection. In the latest official commentary, 21,456 operations were cancelled at the last minute in the third quarter (Q3) 2025/26, and 4,821 patients (22.5%) breached the 28-day standard. Recent provider files identify only a small number of independent-sector organisations. Across the public extracts reviewed from 2021/22 to 2025/26 (Q1-Q3), identifiable independent-sector providers accounted for 296 cancellations versus 337,004 in NHS organisations, contributing under 0.2% of recorded cancellations in each year examined. However, the public files do not provide matched provider-level denominators or case-mix adjustment, and the reporting scope has changed over time. The main conclusion is therefore methodological: current public English data are suitable for surveillance of NHS-funded last-minute cancellations, but they do not permit a fair denominator-matched comparison of day-of-surgery cancellation rates between NHS trusts and private providers. Future comparative work will require linked activity denominators, transparent provider classification, and standardised sector-wide reporting.

## Introduction and background

Last-minute cancellation is defined in NHS England guidance as cancellation on or after the day of admission, including the day of surgery, for non-clinical reasons. The NHS Constitution states that patients whose operations are cancelled for non-clinical reasons should be offered another binding date within 28 days or funded treatment at the time and hospital of the patient’s choice [1-3]. A ‘28-day breach’ refers to failure to provide a new treatment date within 28 days of cancellation.

For surgeons, anaesthetists, service managers, and trainees, cancellation rates matter because they are a visible marker of theatre productivity, inpatient bed and critical-care capacity, preoperative pathway quality, and patient experience. At a national level, NHS England publishes a long-running cancelled elective operations time series beginning in 1994/95 together with provider-level data and quarterly commentary [1,2,4-7].

The policy context is also relevant. NHS-funded elective activity has increasingly been delivered in the independent sector during elective recovery, yet the broader consequences for outcomes, training, workforce distribution, and case-mix remain debated. A recent rapid narrative review concluded that independent-sector providers can deliver high-volume, lower-complexity elective care in some contexts, but that system effects are heterogeneous and the evidence base remains limited [4].

This review aimed to map the publicly available evidence on day-of-surgery or last-minute elective surgery cancellations in England, with specific attention to whether the currently available data allow meaningful comparison between NHS trusts and private or independent-sector providers.

## Review

Methods

This was a targeted narrative review rather than a formal systematic review. Publicly available NHS England cancelled operations webpages, commentary documents, annual provider extracts, and the downloadable historical time-series workbook were reviewed. Targeted searches were conducted using PubMed and web-based sources using terms including ‘surgery cancellation’, ‘elective surgery NHS’, and ‘independent sector healthcare UK’. Public NHS England datasets were identified via the official statistics portal. Sources were included if they reported non-clinical cancellations or system-level analyses.

For the provider-level comparison, the annual NHS England Quarterly Monitoring of Cancelled Operations (QMCO) extracts for 2021/22 (Q3 onwards), 2022/23, 2023/24, 2024/25, and 2025/26 (Q1-Q3) were reviewed [1,2,5,7]. Independent-sector providers were identified directly from organisation names appearing in those files.

Multiple NHS England datasets and documents (including quarterly statistical commentaries, provider-level extracts, and historical time-series files) were reviewed, alongside 13 supporting peer-reviewed and policy sources. These materials are cited using consolidated references where appropriate, rather than as individual entries for each dataset. The NHS England material comprised the main cancelled-operations data landing page, the historical time-series workbook, five recent annual/provider extract files, and three statistical commentary documents. Because the public QMCO files do not provide matched provider-level denominators for the number of scheduled operations, the comparison is descriptive and should not be interpreted as a risk-adjusted sector comparison.

Results

Overview of Publicly Available Data Sources

The key national source is the NHS England Cancelled Elective Operations collection. The public webpage provides a national time series from 1994/95 to the present, quarterly commentary, and provider-level quarterly or annual extracts [1,2,5,6,7]. These sources support longitudinal surveillance but were not designed primarily for cross-sector benchmarking.

A major caveat is that the scope of reporting has not been fully stable over time. Earlier guidance defined a provider broadly enough to include independent-sector organisations, and older provider files explicitly reported England totals both excluding and including the independent sector. In contrast, the latest official commentary for Q3 2025/26 states that its headline summary covers NHS providers excluding independent-sector organisations [2,5, 8-10]. This shift complicates temporal comparison and weakens any simple NHS-versus-private narrative. Table 1 provides a comparison of publicly available English data sources relevant to NHS versus independent-sector.

**Table 1:** Publicly available English data sources relevant to NHS versus independent-sector comparisons This table has been created by the authors from the cited public NHS England policy and literature sources [1,2,4,5,6,7]. PDF: Portable Document Format; QMCO: Quarterly Monitoring of Cancelled Operations

Data source	Coverage	What it contains	Main limitation for sector comparison
NHS England time-series workbook [1]	England, quarterly, 1994/95–present	Cancelled operations, 28-day breaches, national elective admissions, national percentages	No provider-level denominators by sector
NHS England provider extracts [1,2,5,6,7]	England, provider level, 2003/04–present; annual files available for recent years	Counts of cancellations and breaches by the organisation	Very limited identifiable independent-sector coverage; mostly counts only
NHS England commentary PDFs [2,5]	Quarterly national summaries	Headline national cancellation and breach percentages	Recent releases exclude independent-sector organisations from the headline summary
Peer-reviewed clinical audits [10]	Selected hospitals or NHS trusts	Broader on-the-day cancellation estimates with reasons	Not directly comparable with routine QMCO definitions
Independent-sector policy and review literature [4,9]	England	Context on outsourcing, data quality, and case-mix	Sparse direct cancellation-rate evidence and heterogeneous methods

A second major limitation is denominator availability. The time-series workbook contains national elective admissions and national cancellation percentages, but provider extracts mainly provide counts of cancellations and 28-day breaches rather than provider-level procedure denominators. Publicly available data, therefore, permit comparison of recorded counts, not robust comparison of sector-specific cancellation rates.

National Trends in Cancellations and 28-Day Breaches

**Figure 1:**
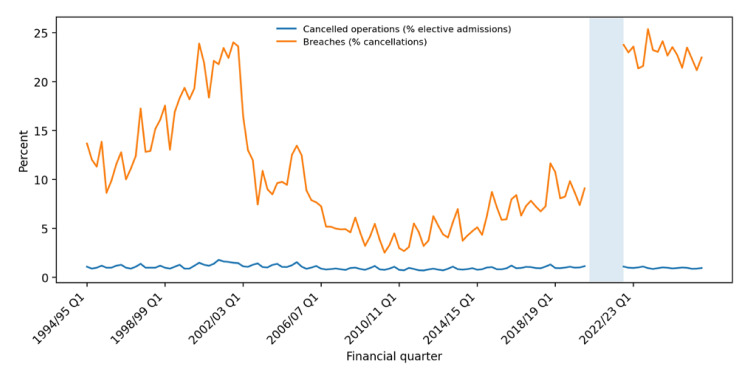
Long-term national trend in cancelled elective operations and 28-day breaches in England The lower line shows last-minute cancelled elective operations as a percentage of elective admissions; the upper line shows the percentage of cancelled patients who were not treated within 28 days of cancellation (28-day breaches). The shaded interval marks the COVID-19 pause in routine data collection from Q4 2019/20 to Q2 2021/22. This figure has been created by the authors in Microsoft Excel (Microsoft Corp., Redmond, WA, USA) using data extracted from the NHS England cancelled elective operations time-series workbook and accompanying statistical commentaries [1,2,5].

Figure 1 shows the long-term national trend in last-minute cancelled elective operations and 28-day breaches, while Table 2 summarises recent provider-level counts derived from the public extracts [1,2,5,7]. The historical time series suggests that cancellation rates rose before the 2002 policy on treatment within 28 days after cancellation, then fell and remained broadly around 1% of elective admissions for much of the next two decades [1,6].

**Table 2:** Recorded cancellations and breaches in recent NHS England provider extracts This table has been created by the authors using NHS England Quarterly Monitoring of Cancelled Operations (QMCO) provider-level files and commentary [1,2,5,7]. Counts are directly obtained from, or summed across, the cited public datasets; the independent-sector share of total cancellations is an author-derived calculation. Note: 2021/22 resumed from Q3 onward after the COVID-19 pause. These figures describe counts rather than risk-adjusted rates because matched provider-level denominators were not available in the public files.

Year	Independent-sector cancellations	NHS cancellations	Independent-sector breaches	NHS breaches	Independent sector share of total cancellations
2021/22 (Q3–Q4 only)	33	36,867	10	8,620	0.09%
2022/23	130	77,266	32	17,698	0.17%
2023/24	43	76,625	12	17,741	0.06%
2024/25	47	85,333	11	19,409	0.06%
2025/26 (Q1–Q3)	43	60,913	3	13,398	0.07%

Quinn et al. likewise found that the 2002 penalty policy was associated with a rapid reduction in cancellation rates below 1%, whereas the 2008 recession and the COVID-19 period more strongly affected breach rates than the cancellation rate itself [6]. The latest NHS England statistical commentary reported 21,456 last-minute non-clinical cancellations in Q3 2025/26, equivalent to 1.0% of elective activity in NHS providers, with 4,821 patients (22.5%) not treated within 28 days [5].

By contrast, in Q3 2019/20, there were 23,503 cancellations in England excluding the independent sector and 2,138 breaches, corresponding to a 9.1% breach rate [7]. The headline cancellation percentage has therefore remained relatively stable, whereas the proportion breaching the 28-day standard has worsened materially since the pandemic-era pause in reporting. This interpretation is consistent with Nuffield Trust analysis, which notes that post-cancellation 28-day breaches have remained well above pre-pandemic levels in the resumed data era [8].

Independent-Sector Representation in Provider-Level Data

Table 3 details the independent-sector organisations identifiable in the recent public QMCO files [1,2,5,7]. Only a very small number of named independent-sector organisations appear consistently in the provider extracts. Across annual files from 2021/22 through 2025/26 (Q1-Q3), these included Kent Institute of Medicine and Surgery (KIMS) Hospital (Newnham Court), Practice Plus Group Surgical Centre-Gillingham, Nuffield Health Cambridge Hospital, iSIGHT, and Practice Plus Group Ophthalmology-North West. In the older Q3 2019/20 provider workbook, The Croft Shifa Health Centre also appeared [7].

**Table 3:** Independent-sector organisations identifiable in recent public Quarterly Monitoring of Cancelled Operations (QMCO) files This table has been created by the authors from public QMCO provider files [1,2,5,7]; it has not been reproduced from a single external source.

Provider name	Recorded cancellations (2021/22 to 2025/26 Q3)	Recorded breaches
Kent Institute of Medicine and Surgery (KIMS) Hospital (Newnham Court)	79	27
Practice Plus Group Surgical Centre-Gillingham	73	25
iSIGHT	86	16
Nuffield Health Cambridge Hospital	57	0
Practice Plus Group Ophthalmology-North West	1	0
The Croft Shifa Health Centre	0	0 (2019/20 Q3 only)

These providers contribute very small absolute numbers of recorded cancellations. In the public extracts reviewed here, independent-sector providers accounted for 33 cancellations in 2021/22 (Q3-Q4 only), 130 in 2022/23, 43 in 2023/24, 47 in 2024/25, and 43 in 2025/26 (Q1-Q3), representing under 0.2% of all recorded cancellations in each year (Table 2).

These counts should not be over-interpreted as evidence that the independent sector has a lower cancellation rate. More plausible explanations are incomplete capture of independent-sector organisations in the public files, narrower and generally lower-complexity case-mix, ring-fenced elective pathways, and the absence of directly comparable provider-level denominators. Ring-fenced elective pathways refer to surgical services where beds, staff, and operating theatres are reserved specifically for planned (elective) operations, so they are not interrupted by emergency cases. Historical concerns about data quality in independent-sector treatment centres have also been noted in policy reviews [4,9].

Discussion

Interpretation in the Context of Existing Literature

Routine NHS England statistics record only last-minute non-clinical cancellations, which is a substantially narrower construct than all day-of-surgery cancellations. This distinction is central to interpreting the literature. Postponement refers to delays prior to the day of surgery and is distinct from same-day cancellation. In the large seven-day prospective cohort study by Wong et al., 13.9% of adult inpatient operations were cancelled on the day of surgery, and non-clinical reasons, especially inadequate bed capacity, accounted for a large share [10]. That figure is far higher than the approximately 1% seen in national QMCO data because the study used a different denominator and a broader definition.

An editorial accompanying that work argued that same-day cancellations are best understood as a system-wide problem spanning bed management, critical-care access, emergency flow, and theatre planning, rather than as an isolated theatre-scheduling problem [11-13]. A systematic review by Koushan et al. similarly found that hospital-related factors such as unavailable theatre time, lack of beds, and scheduling failures commonly dominate elective surgery cancellations internationally [14]. These findings support the interpretation that relatively stable national NHS cancellation percentages can coexist with major variation in avoidable pathway inefficiency and post-cancellation delays.

More recent English perioperative-pathway work also indicates a substantial burden before the day of surgery itself. In 16 NHS trusts, McCone et al. found 583 postponements among 8,000 reviewed case notes (7.3%), with wide inter-trust variation from 1.0% to 31.9% [12]. Quality-improvement work in preoperative assessment has likewise shown that better workup and communication can reduce avoidable same-day disruption [15,16]. Taken together, this literature suggests that national last-minute non-clinical cancellation returns capture only one part of a wider continuum of perioperative unreliability.

Implications for NHS Versus Independent-Sector Comparison

The main practical conclusion is that a fair public comparison between NHS trusts and private providers is not currently possible using routine English data alone. Three methodological problems recur. First, the capture of independent-sector organisations is incomplete and appears to have changed over time [1,5,7]. Second, the QMCO extracts lack provider-level denominators for scheduled procedures or elective admissions, preventing calculation of sector-specific cancellation rates. Third, the available public datasets do not adjust for case mix, urgency, day-case proportion, protected elective-site status, or the fact that outsourced activity may preferentially involve lower-complexity pathways [4,9].

These limitations matter because NHS trusts and independent providers do not operate under identical conditions. NHS hospitals more often carry emergency care, critical-care demand, complex cancer surgery, training responsibilities, and acute bed pressures. Independent providers more commonly operate ring-fenced elective pathways with limited exposure to unscheduled admissions. Quinn et al. found that breaches of the 28-day standard were especially affected in trusts with emergency departments, reinforcing the view that acute-pressure exposure is a key driver of elective disruption [6].

Accordingly, the most defensible interpretation of the current public data is not that one sector categorically performs better than the other, but rather that public reporting has not yet been configured for like-for-like benchmarking. A stronger comparative analysis would require mandatory inclusion of all NHS-funded providers, stable provider classification across time, matched denominators, and at least basic case-mix adjustment.

Practical and Training Implications

For a surgery or anaesthesia readership, the data are still useful even without a definitive cross-sector rate comparison. First, they clarify the difference between routine national last-minute non-clinical cancellations and the broader construct of same-day clinical and non-clinical cancellations reported in audit studies. Second, they show that the post-pandemic deterioration in 28-day treatment after cancellation may be more policy-relevant than small quarter-to-quarter changes in the headline cancellation percentage.

Third, the findings have implications for training environments. If lower-complexity elective work is increasingly shifted to ring-fenced independent-sector pathways, NHS trusts may be left with a more complex residual elective and emergency case mix. That could influence not only cancellation patterns but also training opportunities, perioperative workload, and staffing requirements, issues raised in the wider independent-sector literature [4].

A useful conceptual framing for trainees is to think of cancellation burden at three levels: (1) routine national last-minute non-clinical cancellations; (2) broader day-of-surgery cancellations captured in clinical audits; and (3) earlier postponements arising from preoperative pathway inefficiency. This layered approach helps reconcile apparently discrepant figures across studies and may be especially relevant to anaesthetic and surgical quality-improvement work.

Limitations of This Review and Future Directions

This review relied on publicly available data and selected literature rather than confidential contract-level information or linked hospital episode statistics. Provider classification in the public files was based on organisation names and may miss independent-sector activity embedded within other commissioning or contracting arrangements. The review also focused on England; data systems in Scotland, Wales, and Northern Ireland are not directly comparable. Independent-sector cancellation data likely represent under-reporting due to incomplete capture in public datasets.

As a narrative review, this article does not claim the comprehensiveness or formal risk-of-bias assessment of a systematic review. Nevertheless, it adds value by explicitly mapping what the public data can and cannot support. Future comparative studies should combine linked provider-level activity denominators with richer information on procedure type, complexity, urgency, anaesthetic requirements, and protected-site status. A parallel policy priority is clearer and more stable publication of NHS-funded activity across both NHS and non-NHS providers.

## Conclusions

Publicly available English data show that last-minute elective cancellation rates have remained close to 1% nationally for many years, but the proportion of cancelled patients not retreated within 28 days rose substantially after COVID-19 and remains well above pre-pandemic levels. Although a small number of independent-sector organisations are visible in recent public provider extracts, they account for a very small share of recorded cancellations, and the public datasets do not provide the denominators needed for a fair rate comparison with NHS trusts. The most important finding is therefore methodological: current public reporting is suitable for surveillance, but not for robust NHS-versus-private benchmarking. Future comparative work should use linked provider-level activity denominators, case-mix adjustment, transparent provider classification, and mandatory reporting across all NHS-funded providers.
